# Splicing Modulation via Antisense Oligonucleotides in Recessive Dystrophic Epidermolysis Bullosa

**DOI:** 10.3390/ijms25020761

**Published:** 2024-01-07

**Authors:** Stefan Hainzl, Lisa Trattner, Bernadette Liemberger, Johannes Bischof, Thomas Kocher, Michael Ablinger, Alexander Nyström, Astrid Obermayer, Alfred Klausegger, Christina Guttmann-Gruber, Verena Wally, Johann W. Bauer, Josefina Piñón Hofbauer, Ulrich Koller

**Affiliations:** 1 EB House Austria, Research Program for Molecular Therapy of Genodermatoses, Department of Dermatology and Allergology, University Hospital of the Paracelsus Medical University, 5020 Salzburg, Austria; s.hainzl@salk.at (S.H.); l.trattner@crcs.at (L.T.); b.liemberger@crcs.at (B.L.); j.bischof@salk.at (J.B.); t.kocher@salk.at (T.K.); m.ablinger@salk.at (M.A.); a.klausegger@salk.at (A.K.); c.gruber@salk.at (C.G.-G.); v.wally@salk.at (V.W.); j.d.pinon@salk.at (J.P.H.); 2 Department of Dermatology, Medical Faculty, Medical Center—University of Freiburg, 79110 Freiburg, Germany; alexander.nystroem@uniklinik-freiburg.de; 3 Core Facility of Electron Microscopy, Department of Environment & Biodiversity, Paris Lodron University Salzburg (PLUS Salzburg), 5020 Salzburg, Austria; astrid.obermayer@plus.ac.at; 4 Department of Dermatology and Allergology, University Hospital of the Paracelsus Medical University, 5020 Salzburg, Austria; joh.bauer@salk.at

**Keywords:** *COL7A1*, antisense oligonucleotides, splicing modulation, recessive dystrophic epidermolysis bullosa

## Abstract

Antisense oligonucleotides (ASOs) represent an emerging therapeutic platform for targeting genetic diseases by influencing various aspects of (pre-)mRNA biology, such as splicing, stability, and translation. In this study, we investigated the potential of modulating the splicing pattern in recessive dystrophic epidermolysis bullosa (RDEB) patient cells carrying a frequent genomic variant (c.425A > G) that disrupts splicing in the *COL7A1* gene by using short 2′-O-(2-Methoxyethyl) oligoribo-nucleotides (2′-MOE ASOs). *COL7A1*-encoded type VII collagen (C7) forms the anchoring fibrils within the skin that are essential for the attachment of the epidermis to the underlying dermis. As such, gene variants of *COL7A1* leading to functionally impaired or absent C7 manifest in the form of extensive blistering and wounding. The severity of the disease pattern warrants the development of novel therapies for patients. The c.425A > G variant at the *COL7A1* exon 3/intron 3 junction lowers the efficiency of splicing at this junction, resulting in non-functional C7 transcripts. However, we found that correct splicing still occurs, albeit at a very low level, highlighting an opportunity for intervention by modulating the splicing reaction. We therefore screened 2′-MOE ASOs that bind along the *COL7A1* target region ranging from exon 3 to the intron 3/exon 4 junction for their ability to modulate splicing. We identified ASOs capable of increasing the relative levels of correctly spliced *COL7A1* transcripts by RT-PCR, sqRT-PCR, and ddPCR. Furthermore, RDEB-derived skin equivalents treated with one of the most promising ASOs exhibited an increase in full-length C7 expression and its accurate deposition along the basement membrane zone (BMZ).

## 1. Introduction

RNA-based therapies hold great potential in addressing defects in many severe genetic diseases, including the blistering skin disease epidermolysis bullosa (EB), spinal muscular atrophy (SMA), and Duchenne muscular dystrophy (DMS) [1]. These therapies can target different aspects of mRNA biology, from its maturation to its degeneration, and thereby present multiple opportunities to not only modulate gene expression, but also tweak the genetic code before it is translated into functional proteins [2]. Several different therapies are currently in development, ranging from RNAi-mediated gene regulation, CRISPR/Cas-based genome editing, spliceosome-mediated RNA *trans*-splicing (SMaRT), and RNA aptamers to modified mRNA molecules for vaccines [3,4,5]. Among these, antisense oligonucleotides (ASOs) have emerged as promising therapeutics for tackling genetic diseases at the RNA level [6], showing considerable potential in fine-tuning gene expression by modulating the splicing process. During the course of mRNA maturation, the process of pre-mRNA splicing can be disrupted by genetic variants in pivotal splicing elements, such as donor and acceptor splice sites. These mutations can lead to aberrant splicing and the generation of a defective RNA molecule, culminating in functional impairment of the resulting protein. By binding to either critical donor/acceptor splice sites or exonic splicing enhancers (ESEs)/intronic splicing silencers (ISSs), ASOs can mediate the exclusion or inclusion of targeted exons. For this reason, ASOs are currently in the developmental pipeline for the treatment of various single-gene disorders [7]. Initial studies utilizing unmodified ASOs to target RNA showed only moderate success due to their vulnerability to degradation by nucleases. However, advancements in ASO research have ushered in a new generation of chemically modified ASOs [8,9,10] with enhanced binding capabilities, an increased stability against enzymatic breakdown, and a reduced potential for triggering immune responses. As a result, these refined ASOs have found diverse applications in addressing numerous diseases, including hereditary transthyretin amyloidosis (hATTR), hypercholesterolemia, chronic hepatitis C, SMA, β-thalassemia, and EB [11]. 

EB is a genetic skin fragility disorder characterized by wound formation even after minor skin trauma. More than 16 genes have been identified as involved in the development of EB [12]. The recessive dystrophic subtype RDEB is caused by gene variants of the *COL7A1* gene, encoding type VII collagen (C7). C7 is a critical component of anchoring fibril suprastructures, maintaining cohesion between the dermal and epidermal layers of the skin [13]. RDEB can present itself as a localized or intermediate form and frequently features more severe symptoms. These include extensive skin blistering, fibrosis, scarring, joint contractures, and systemic complications like anemia, osteoporosis, or malnutrition [14]. 

**Figure 1:**
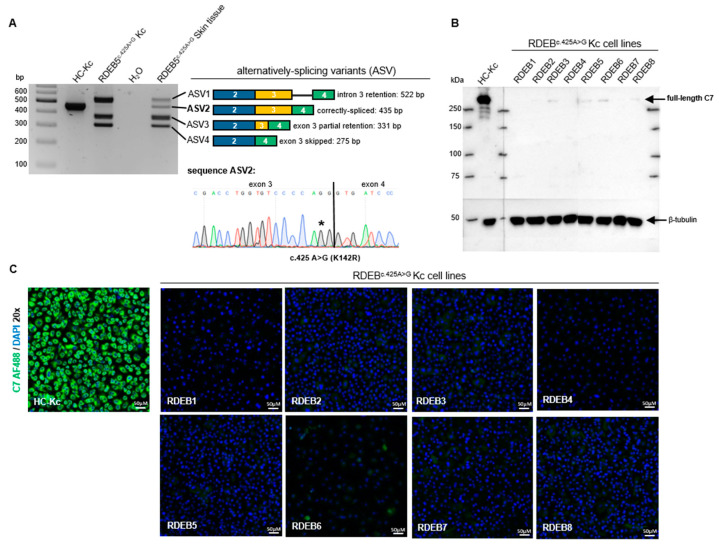
Characterization of the *COL7A1* splicing pattern and C7 expression in RDEB patients carrying the c.425A > G (p.K142R) missense variant. (**A**) Residual levels of correctly spliced *COL7A1* (ASV2) were detected in keratinocytes and skin tissue samples from an RDEB patient (RDEB5) carrying the homozygous c.425A > G (p.K142R) variant on the genomic level. * marks the exchanged base (from A to G) on position 425 in the ASV2 variant. (**B**) Western blot analyses confirmed the presence of residual full-length C7 at 290 kDa. β-tubulin (~50 kDa) was used as a loading control. (**C**) Immunofluorescence staining revealed varying levels of C7 expression (green), correlating with the Western blot results. HC-Kcs showed robust C7 protein levels, whereas RDEB5^c.425A>G^ Kcs displayed minimal or absent C7 levels. The cells’ nuclei were stained with 4′,6-Diamidino-2-phenylindol (DAPI, blue). Scale bar = 50 µm.

A significant subset of RDEB patients carries a specific genomic variant c.425A > G that disrupts splicing within the *COL7A1* gene [15]. This variant reduces the efficiency of the donor splice site at the *COL7A1* pre-mRNA exon 3/intron 3 junction. This allows alternative splicing reactions to take place that result either in the joining of exon 2 directly to exon 4 (exon 3 exclusion), the use of a cryptic splice site within exon 3, or the retention of intron 3 [16]. These alternative splicing events result in the coded message running into a pre-termination codon (PTC). While the splicing outcomes of this variant were originally documented by Gardella et al. in 1996 [16], our recent research has revealed the presence of residual levels of correctly spliced *COL7A1* mRNA in samples taken from several individuals with RDEB (Figure 1). In this context, we sought to leverage ASO technology to target the region of the c.425A > G variant, with the goal of boosting correct *COL7A1* exon 3/intron 3 splicing in order to obtain full-length C7 protein expression.

Notably, the above concept relies on an ASO-mediated exon-retention strategy rather than the more widely applied exon-skipping strategy. Nonetheless, there is precedent for the success of such an exon-retention strategy, as marked by the development and US Food and Drug Administration (FDA) approval of Nusinersen (marketed as Spinraza^®^) for treating SMA [17]. This debilitating neuro-muscular condition, afflicting approximately 1 in 10,000 individuals, predominantly arises from the homozygous deletion of exon 7 in the *SMN1* gene [18]. Humans carry a second gene, *SMN2*, which bears 99% homology to *SMN1* [19]. However, *SMN2* does not contribute to the pool of SMN proteins due to an exonic splice enhancer mutation that causes exon 7 to be predominantly skipped, resulting in a truncated, non-functional protein [20]. Spinraza^®^ deploys ASOs as synthetic splice enhancers to promote the inclusion of exon 7 in the *SMN2* transcript to generate a fully functional SMN protein [7]. Additionally, Breuel et al. identified a variant within the *BBS1* gene (c.479G > A) responsible for exon 5 skipping and intron 5 retention, a genetic anomaly associated with Bardet–Biedl syndrome [21]. Their inventive approach combined ASOs with an engineered U1 small nuclear RNA (snRNA), yielding an improved therapeutic efficacy for genomic-variant-induced splicing defects within the *BBS1* gene. 

Given the remarkable similarities between the splicing defects in Bardet–Biedl syndrome (BBS), SMA, and RDEB, efforts in these diseases offer invaluable insights for the development of ASO-based therapies tailored to the specific splicing abnormality mediated by the *COL7A1* c.425A > G variant in RDEB. Here, we describe our meticulous efforts to develop such a strategy, thereby highlighting the potential to partially revert the *COL7A1* splicing profile in RDEB cells.

## 2. Results

### 2.1. Characterization of the Splicing Pattern in RDEB Keratinocytes

A critical contributor to the pathogenesis of RDEB is the homozygous c.425A > G variant in exon 3 of *COL7A1* [16]. This genomic variant is found in approximately 12.8% of all RDEB patients in central Europe [15]. Due to the frequency of the mutation within the cohort of RDEB patients at the EB House in Salzburg, we analyzed the splicing consequences of this mutation in more detail.

An initial splicing pattern analysis via reverse transcription (RT)-PCR revealed the presence of four alternative splicing variants (ASVs) (Figure 1A), including all three variants previously identified by Gardella et al.: intron 3 retention (ASV1), partial exon 3 retention (ASV3), and exon 3 skipping (ASV4) [16]. In ASV3, the first 56 nucleotides (nts) of exon 3 are fused to exon 4, possibly due to the presence of an alternative donor splice site within exon 3. In contrast, ASV2 includes the accurate exon 3/exon 4 junction but encodes the A > G mutation at transcript position 425 compared to the wild-type transcript. Notably, correctly spliced *COL7A1* transcripts (ASV2) were detected in one skin tissue sample and in six out of eight primary keratinocyte cultures (Supplementary Figure S1) established from RDEB patients that were either homozygous (three out of eight) or heterozygous (five out of eight) for the c.425A > G variant on the genomic level (henceforth referred to as RDEB^c.425A>G^; Figure 1A and Table 1). Western blot analyses of protein lysates from healthy control (HC) keratinocytes (HC-Kcs) showed a robust C7 expression, detectable at 290 kDa. In contrast, primary RDEB^c.425A>G^ keratinocytes (RDEB^c.425A>G^ Kcs) showed only residual C7 levels in five out of eight cell lines (Figure 1B). Notably, five RDEB patients included in the study are combined heterozygous for the c.425A > G variant. In general, this genotype has an impact on the level of protein expression. However, in our case, all analyzed primary RDEB keratinocyte cultures express only negligible amounts or no C7, as verified via Western blot analyses (Figure 1B) and immunofluorescence staining (Figure 1C). Thus, the genomic variants encoded in the second *COL7A1* allele in these cells do not appear to result in C7 expression. 

**Figure 2:**
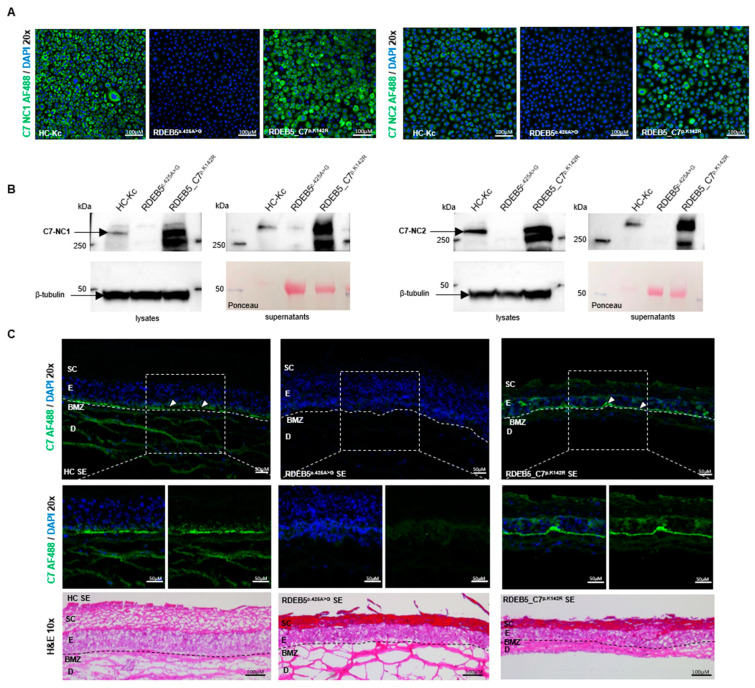
Analysis of C7 expression and localization in RDEB5^c.425A>G^ Kcs and an RDEB-derived 3D organotypic skin model. (**A**) Immunofluorescence analysis of RDEB5^c.425A>G^ retrovirally transduced with the mutation-carrying full-length *COL7A1* revealed a pronounced overexpression of C7 when stained with NC1 and NC2 antibodies (green). By contrast, untransduced RDEB5^c.425A>G^ Kcs showed no detectable C7 expression. Cells’ nuclei were counterstained with 4′,6-Diamidino-2-phenylindole (DAPI, blue). Scale bar = 100 µm. (**B**) Western blot analyses on cell lysates and cell culture supernatants confirmed the full-length expression and secretion of C7^p.K142R^ at 290 kDa. HC-Kcs served as a positive control and RDEB5^c.425A>G^ Kcs as a negative control. (**C**) Immunofluorescence analysis conducted on cryosections showed elevated levels of C7 protein (green) localized at the BMZ in SEs generated from C7^p.K142R^-overexpressing RDEB5^c.425A>G^ Kcs. SEs expanded from HC-Kcs and fibroblasts demonstrated natural levels of C7 expression at the BMZ absent in RDEB5^c.425A>G^-derived SEs. The formation and organization of SEs were confirmed by H&E staining. Cells’ nuclei were stained with DAPI (blue). SC: stratum corneum, E: epidermis, BMZ: basement membrane zone, D: dermis. Scale bar = 50 µm. Images show 3 × 2 tile scans of the 20x objective.

Accurate splicing of *COL7A1* in RDEB^c.425A>G^ (i.e., ASV2) is predicted to result in the expression of the full-length C7 protein carrying a single conservative amino acid exchange (p.K142R) (Supplementary Figure S2A) [22]. This exchange is not expected to significantly alter protein folding, stability, or function. In order to confirm this, we generated a retroviral vector containing the full-length cDNA of *COL7A1*, including the respective missense variant (c.425A > G) within exon 3. The viral vector was transduced into RDEB5^c.425A>G^ Kcs to achieve overexpression of C7^p.K142R^ (RDEB5_C7^p.K142R^ Kcs) and thus the likeliness of obtaining detectable levels of accurately spliced C7. Immunofluorescence staining showed strong expression levels of C7^p.K142R^ in RDEB5_C7^p.K142R^ Kcs (Figure 2A). Western blot analysis revealed the expression of full-length C7^p.K142R^ and its secretion into the cell culture medium (Figure 2B), as well as the formation of C7 trimers in the cells (Supplementary Figure S2B). To determine whether C7^p.K142R^ protein localization within the basement membrane zone (BMZ) resembled that of the wild-type protein, we generated 3D organotypic skin equivalents (SEs) using these modified cells (Figure 2C). While SEs grown from parental RDEB5^c.425A>G^ Kcs and RDEB5^c.425A>G^ fibroblasts showed no C7 expression, SEs derived from RDEB5_C7^p.K142R^ keratinocytes and RDEB5^c.425A>G^ fibroblasts showed accurate deposition of C7^p.K142R^ within the BMZ. These results demonstrate the accurate expression and deposition of C7^p.K142R^ and set the basis for the development of ASOs that can enhance the levels of accurate splicing at the exon 3/intron 3 junction as a viable strategy to increase functional C7 levels in RDEB^c.425A>G^ patients. 

### 2.2. Initial Screening Identifies ASOs Capable of Modulating Splicing in RDEB Keratinocytes

**Figure 3:**
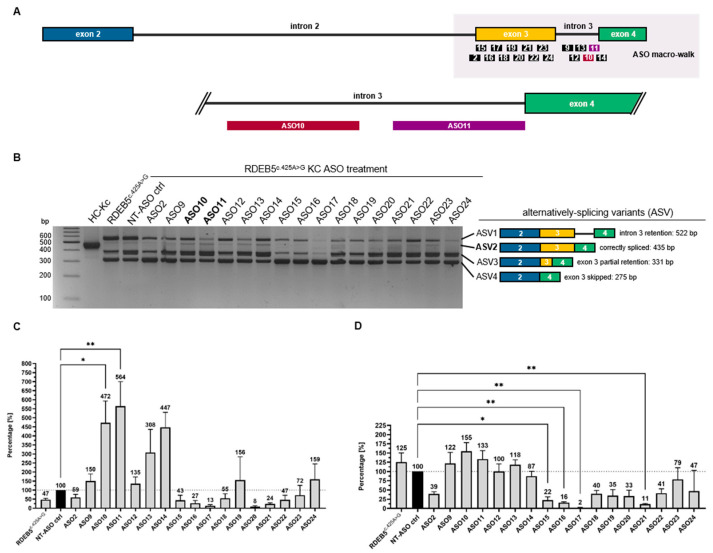
Evaluating the impact of ASOs on *COL7A1* splicing: identifying key binding sites and effects on exon 3 inclusion. (**A**) Schematic presentation of the binding positions for the ASOs employed in the initial macro-walk study. (**B**) Transfection of RDEB5^c.425A>G^ Kcs with 600 nM of individual ASOs led to various consequences in the *COL7A1* splicing pattern. The cDNA of HC-Kcs and untreated, as well as NT-ASO control-treated, RDEB5^c.425A>G^ Kcs served as positive and negative controls, respectively. The rate of exon 3 inclusion in each sample was determined using Fiji (ImageJ) software (Fiji version number: ImageJ 2.1.0; Java 1.8.0_172). (**C**) ASO10 and ASO11 treatment resulted in the highest rate of ASV2 generation. (**D**) Exon 3 inclusion was further evaluated by sqPCR analysis, indicating the up- (e.g., ASO10 and ASO11; 1.55- and 1.33-fold, respectively) and downregulation (e.g., ASO 2, 15, 16, 17, and 21) of accurate *COL7A1* transcripts in certain ASO-treated RDEB5^c.425A>G^ Kcs. Densitometric analysis *n* = 3; sqPCR analysis *n* = 5; mean = ±SEM. *p*-values (significances): >0.05 (not significant), ≤0.05 (*), ≤0.01 (**).

We postulated that short ASOs can interact with as yet unknown splicing elements (e.g., ESEs) in proximity to the c.425A > G mutation site, promoting the usage of the 5′ donor splice (exon 3/intron 3) site and ultimately leading to the incorporation of exon 3 in the final transcript (i.e., correct splicing). We therefore performed an initial screening of 17 overlapping 2′-MOE ASOs, each 20 nts in length (Supplementary Table S1). These ASOs bind along the 267-nucleotide *COL7A1* target region, ranging from exon 3 to the intron 3/exon 4 junction (Figure 3A). We specifically screened for ASOs capable of increasing the level of correctly spliced *COL7A1* transcripts in RDEB5^c.425A>G^ Kcs. 

After performing a concentration kinetics analysis (Supplementary Figure S3A,B), we initially transfected the target keratinocytes with the different ASOs at a concentration of 600 nM using INTERFERin^®^ transfection reagent. Preliminary data using a Cy5-labeled ASO had demonstrated an almost 100% transfection efficiency with this reagent (Supplimentary Figure S3C). Two days post transfection, the total RNA was extracted and analyzed by RT-PCR using primers that amplified the entire target region from *COL7A1* exon 2 to exon 4. Different splicing products were separated by gel electrophoresis and identified based on their predicted molecular weight. Correct identification of ASV2 was verified by Sanger sequencing and further analyzed in SnapGene^®^ Viewer software (Dotmatics; version 2.3.3).

Depending on the ASO transfected, we were able to detect increased levels of correctly spliced *COL7A1* transcripts (ASV2) or modulation (downregulation or upregulation) of the three other remaining cryptic splice products (ASV1, ASV3, ASV4). This combined effect was particularly visible in cells treated with ASO10 and ASO11 (Figure 3B), which bound at the 3′ end of intron 3. A densitometric analysis was performed on the four alternative splicing variants (Supplementary Figure S3D). The amount of correctly spliced product (ASV2) was calculated as a fraction of all splice products (ASV1 + ASV2 + ASV3 + ASV4) present in each treatment (i.e., fractional abundance). The percentage increase in the fractional abundance of ASV2 upon treatment with the different ASOs was calculated with respect to the non-targeting (NT) ASO control (NT-ASO control). These results indicated that ASOs 10 and 11 were the most potent, achieving mean 4.72- and 5.64-fold increases in the levels of correctly spliced *COL7A1* transcript, respectively (Figure 3C). Interestingly, both ASOs additionally upregulated ASV3 and ASV4 when introduced into the target cells in a concentration of 100 nM and 600 nM, respectively (Supplementary Figure S3B).

We additionally designed semi-quantitative real-time PCR (sqPCR) assays to specifically detect the desired C7^p.K142R^-encoding transcript (ASV2) out of all *COL7A1* transcripts present. For the C7^p.K142R^-encoding transcript, we used a (reverse) primer binding exactly to the exon 3^c.425A>G^/exon 4 junction, together with a (forward) primer binding to the exon 1/2 junction. The specificity of this PCR assay was confirmed by Sanger sequencing of the single amplicon generated by the reaction (Supplementary Figure S3E). To quantify the total *COL7A1*-transcripts, we used primers specific for exon 2, which is present in all *COL7A1*-derived transcripts regardless of the ensuing alternative splicing events around exon 3. Again, the amount of ASV2 was calculated as a fraction of the total *COL7A1* transcripts, and the increase in the fractional abundance of ASV2 induced by the different ASOs was normalized to the NT control. While the effect of treatment on splicing modulation was more modest than when evaluated via the method of densitometric analysis, the sqPCR assays confirmed that ASOs 10 and 11 induced the most efficient upregulation of correctly spliced C7^p.K142R^ transcripts (ASO10 and ASO11: 1.55- and 1.33-fold increases, respectively, over the NT-ASO control) (Figure 3D).

### 2.3. ASO Micro-Walk along the ASO10/ASO11 Binding Region

**Figure 4:**
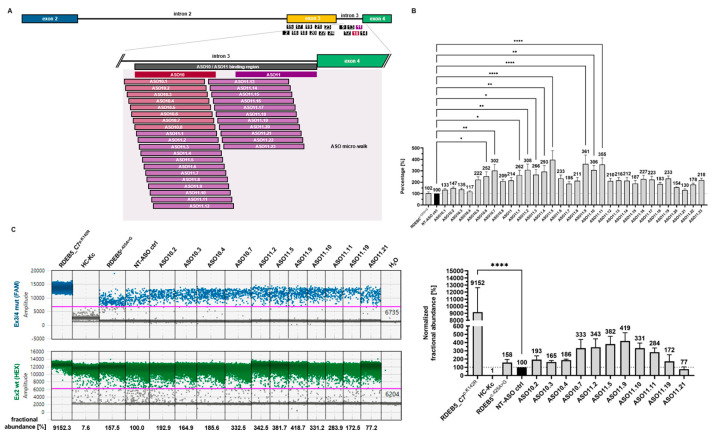
Evaluating the impact of ASOs on *COL7A1* splicing: identifying key binding sites and effects on exon 3 inclusion. (**A**) Schematic presentation of the binding position for the ASOs employed in the micro-walk study. (**B**) sqRT-PCR analysis revealed the upregulation of ASV2 transcripts for ASO10.7 (1.41-fold); ASO11.2 (1.44-fold); ASO11.5 (1.85-fold); ASO11.9 (1.68-fold); ASO11.10 (1.43-fold); and ASO11.11 (2.67-fold) over the NT-ASO control. (**C**) Exon 3 inclusion was further evaluated via a ddPCR analysis, revealing the upregulation of accurately spliced *COL7A1* transcripts in distinct ASO-treated RDEB5^c.425A>G^ Kcs. Asv2 transcripts were upregulated in ddPCR analysis for ASO10.7 (3.35-fold); ASO11.2 (3.43-fold); ASO11.5 (3.82-fold); ASO11.9 (4.19-fold); ASO11.10 (3.31-fold); ASO11.11 (2.84-fold); and RDEB5_C7^p.K142R^ (91.52-fold) over the NT-ASO control. sqPCR analysis micro-walk *n* = 12; ddPCR *n* = 4; mean = ±SEM. *p*-values (significances): >0.05 (not significant), ≤0.05 (*), ≤0.01 (**), ≤0.0001 (****).

Based on the above results, we performed a more comprehensive screening of the 45 bp region targeted by ASOs 10 and 11 (Figure 4A). Given a particular target region, there is currently no algorithm to predict the best performing ASO sequence to achieve the desired splicing modulation. As such, the development of ASO therapeutics typically involves a micro-walk, wherein tested ASOs are shifted by one nucleotide along the target region with respect to each other [23]. We therefore designed 31 individual overlapping ASOs to systematically cover the target region (Supplementary Table S2). ASO efficacies were initially assessed via sqPCR as described above. We observed high variances in the effects of the different ASOs on the levels of the correctly spliced variant ASV2. As also witnessed by other groups, shifting the sequence of an ASO by 1 nt in the 5′ or 3′ direction can have tremendous impacts on its efficacy on splicing modulation [7]. As an example, the binding position of ASO11 differs from that of ASO11.19 by only 1 nt in the 5′ direction, yet treatment with ASO11.19 was associated with a 10% increase in correct splicing compared to ASO11. From this analysis, we identified several ASOs that consistently outperformed the rest in enhancing the levels of correctly spliced C7^p.K142R^ transcripts, namely ASO10.7, ASO11.2, ASO11.5, ASO11.9, ASO11.10, and ASO11.11 (Figure 4B).

Finally, we confirmed these results using a digital droplet PCR (ddPCR) assay designed to quantify the absolute fractional abundance of C7^p.K142R^ transcripts achieved via ASO treatment (Figure 4C). Similar to the sqPCR assays described above, the ddPCR assay was designed to detect C7^p.K142R^ transcripts using a carboxyfluorescein FAM-labeled probe specific for the exon 3^c.425A>G^/exon 4 junction, and total C7-transcripts using a hexachlorofluorescein HEX-labeled probe specific for *COL7A1* exon 2. The fractional abundances of C7^p.K142R^ transcripts achieved were calculated as percentage increases or decreases with respect to treatment with the NT-ASO control. This assay was conducted on the top six performing ASOs detected via sqPCR, as well as four under-performing ASOs in addition to the untreated and NT-ASO control-treated Kcs. Detection of the engineered C7^p.K142R^ transcript from the RDEB5_C7^p.K142R^ cell line was used as a positive control. The results of the ddPCR assay mirrored the sqPCR analysis in identifying ASOs 11.5 and 11.9 as the top performing ASOs. These two ASOs are therefore considered the most suitable for potential future development of this therapeutic strategy for c.425A > G *COL7A1*-caused RDEB. 

### 2.4. Impact of ASO Treatment on C7 Protein Expression

**Figure 5:**
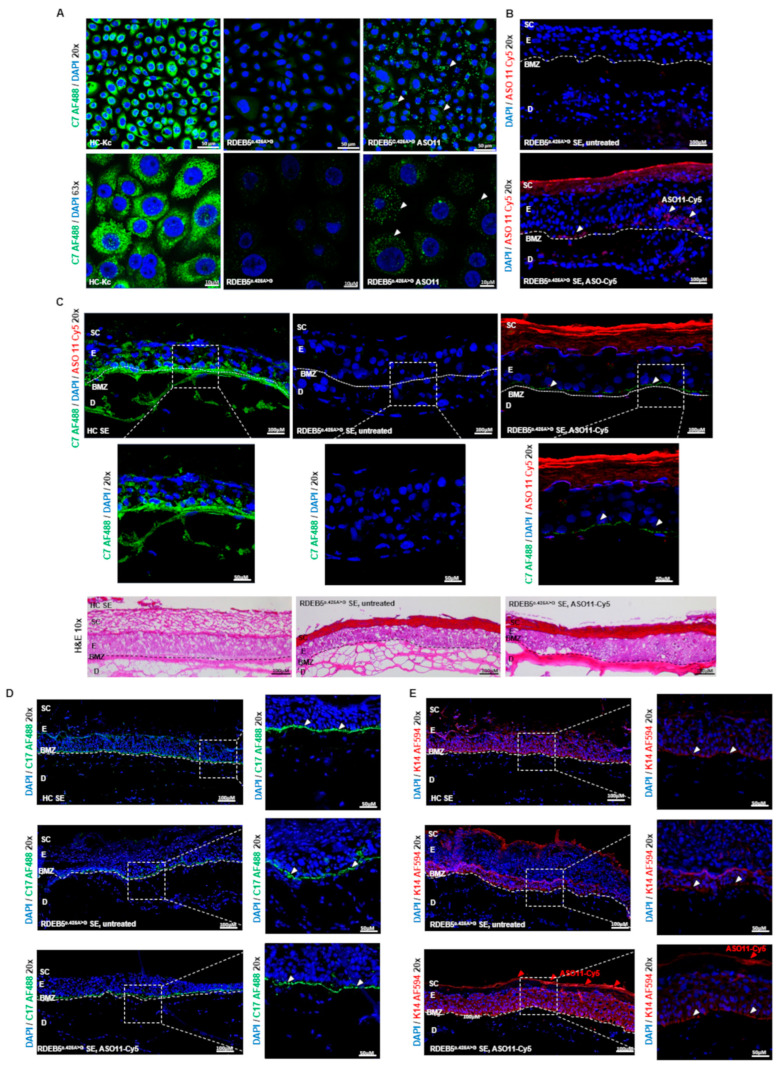
Immunofluorescence analysis of ASO-treated RDEB5^c.425A>G^ Kcs in monolayer cultures and SEs. (**A**) Immunofluorescence analysis of ASO11-treated RDEB5^c.425A>G^ Kcs showed an increased C7 expression (green) compared to untreated RDEB^c.425A>G^ Kcs. Cells’ nuclei were counterstained with 4′,6-Diamidino-2-phenylindole (DAPI, blue). Scale bar (20×) = 50 µm; scale bar (63×) = 10 µm. (**B**) Upon DDC642 liposome-mediated delivery of Cy5-labeled ASO11 into RDEB5^c.425A>G^ SEs, we observed ASO accumulation at the stratum corneum and along the BMZ. (**C**) Immunofluorescence analysis conducted on cryosections of ASO11-treated RDEB5^c.425A>G^ SEs showed increased levels of C7 protein (green) localized at the BMZ in comparison to untreated RDEB5^c.425A>G^ SEs. SEs generated from HC-Kcs and fibroblasts served as a positive control and showed natural expression levels of C7 along the BMZ. Cell nuclei were stained with DAPI (blue). (**D**) Immunofluorescence analysis of C17 expression (green) in SEs. (**E**) Immunofluorescence analysis of K14 expression (red) in SEs. SC: stratum corneum, E: epidermis, BMZ: basement membrane zone, D: dermis. Scale bar (20×) = 100 µm, scale bar (zoom box) = 50 µm. Images B and C show 3 × 2 tile scans of the 20× objective. Images D and E show 6 × 2 tile scans of the 20× objective.

To demonstrate the impact of ASO-mediated modulation of *COL7A1* splicing on the production of the full-length C7 protein, RDEB5^c.425A>G^ Kcs in culture were treated with either ASO11 or NT-ASO control every 24 h for 2 days. C7 expression levels were evaluated via immunofluorescence staining 96 h after the first transfection. We observed a minor but reproducible upregulation of C7 expression in cell monolayers (Figure 5A). We reasoned that evaluation of ASO-mediated restoration of full-length C7 expression can be facilitated in SEs expanded from parental RDEB5^c.425A>G^ Kcs and RDEB5^c.425A>G^ fibroblasts, since functional C7 is deposited over time and can stably accumulate at the interface between the dermis and the epidermis. Thus, we first investigated the correct distribution of the ASOs within the BMZ by treating SEs with Cy5-labeled ASO11 complexed to DDC642 liposomes [24], a carrier previously used in splicing–modulating approaches for JEB [25]. Although most Cy5-labeled ASOs remained at the stratum corneum, we were able to detect Cy5-labeled ASOs along the basal layer of the epidermis within the SE (Figure 5B). Furthermore, we observed an accumulation of C7 as well as type XVII collagen (C17) and Cytokeratin 14 (K14) within the BMZ (Figure 5C–E).

## 3. Discussion

Gene therapeutic strategies for RDEB have mainly focused on gene replacement or gene editing. While gene replacement has already advanced to the clinical stage, gene editing is still at the pre-clinical stage. Until now, gene therapies tested in clinical trials on EB patients have either focused on ex vivo [26,27] or in vivo [28,29] applications (generally summarized in the review of Marinkowich and Tang, 2019 [30], and the clinical perspectives outlined in the review of Welponer et al., 2021 [31]). Recently, the FDA approved an in vivo gene therapeutic drug, Vyjuvek^®^, marking a significant milestone for the treatment of EB [32]. In this topical in vivo gene therapy for dystrophic EB (DEB), a non-replicating herpes simplex virus (HSV) is used to deliver two copies of *COL7A1* cDNA into skin cells persisting in wounds of DEB patients. As a result, 67% of wounds remained closed 6 months after the first application. In comparison, only 22% of placebo-treated wounds remained closed at this time point [29]. Although the approval of Vyjuvek^®^ represents a great achievement in the field of gene therapies for genodermatoses, the development of other non-viral-based therapeutic strategies for RDEB patients is a viable and important strategy. This includes the use of ASOs to revert splicing consequences from splice site mutations in genetic diseases. Several studies have highlighted the potential of ASO-mediated splicing modulation as a key strategy in treating monogenetic diseases [33,34,35]. In the context of RDEB, ASOs have been exploited to modulate the pre-mRNA splicing process to skip hotspot mutation-bearing in-frame exons, such as exons 73, 80, and 105, from the final mRNA [36,37]. The exclusion of these exons generates the possibility of at least partially restoring the function to the protein, thus potentially modifying disease severity. Preliminary studies involving RDEB fibroblasts and keratinocytes with mutations in exons 73 and/or 80 highlighted the potential of exon skipping, aided by 2′-MOE ASOs, to restore type VII collagen expression and the formation of anchoring fibrils [37]. 

Our work here further highlights the therapeutic potential of ASO-mediated splice modulation for RDEB. The effectiveness of exon inclusion has been exemplified in previous studies, notably those conducted by Krainer et al., which have served as the basis for the successful clinical development of Spinraza^®^ for SMA [7,17,38,39,40,41]. The c.425A > G variant in exon 3 of *COL7A1* bears remarkable similarities to the variant targeted by Spinraza^®^, prompting us to initiate this study to test a similar strategy for RDEB. Our findings demonstrate an up to 76% enhancement in ASV2 transcripts in keratinocytes treated with the most potent ASOs identified via the micro-walk, warranting a more in-depth exploration of the therapeutic implications of this strategy. However, to support further clinical development, a more sophisticated disease model for testing would be needed in order to enable a clearer picture of the potential long-term therapeutic outcomes for RDEB patients. A major challenge in this respect is the need for models that not only recapitulate the human genetic defect and the sequences around it, but also accommodate repeated topical administration to mimic the intended route of administration in clinics. A primary obstacle in this respect is still the effective delivery of molecules through the epidermal barrier [42,43,44]. Naturally, ASOs need to enter cells and reach the target RNA to induce their antisense activity [45,46]. In RDEB, the target cells are the basal keratinocytes and dermal fibroblasts in the BMZ, which lies underneath several differentiated epidermal layers, including the stratum corneum. Similar to Vyjuvek^®^, treating open RDEB wounds presents an opportunity to circumvent this problem [47]. However, ASOs still require proper packaging to protect them from degradation and facilitate their successful entry into cells [48]. One potential future delivery platform is a recently developed ionic liquid formulation used to successfully deliver siRNAs to the skin in several pre-clinical animal models [49]. In addition to testing delivery platforms, a significant focus should be put on optimizing the ASO backbone for clinical applications. A thorough examination of different chemical modifications is imperative [11] in order to improve not only the delivery, but also the stability of the ASO [50]. This is especially important in the context of RDEB, where chronic wounds have a persistent inflammatory background. As such, further studies are essential to determine whether the 2′-MOE backbone remains the optimal choice for our intended application [51]. Moreover, it is important to consider RNA secondary structures within the ASO10-11 binding region that may influence or underpin the splice-modulating capabilities of our most promising ASOs. Such secondary structures may sequester important splicing elements and thereby alter splicing patterns, causing disease [52]. As such, ASOs or antisense peptide nucleic acids (asPNAs) can be designed to disrupt potentially inhibitory RNA secondary structures, as has been successfully applied by Ong et al. to modulate disease-associated aberrant tau pre-mRNA alternative splicing [53]. Currently, there are over 100 ongoing clinical trials for ASO-mediated gene therapies [11,54,55,56,57]. Having said that, only one of the trials evaluated the use of a topically applied ASO in the context of DDEB or RDEB. QR-313’s mechanism of action involves binding a specific sequence in the *COL7A1* pre-mRNA, leading to the exclusion of exon 73 [36]. Unfortunately, despite promising results in pre-clinical development, phase I/II trials of QR-313 (Wings Therapeutics) were terminated due to low enrollment (NCT03605069).

In conclusion, this work highlights the potential of ASO-mediated splice modulation for the treatment of EB-related conditions. Our data also emphasize the need for continued exploration and refinement of this technology. Progressing towards unveiling the full therapeutic potential of this method demands addressing challenges related to modifications and delivery as well as confirmation of its efficacy in a diverse array of disease-relevant models.

## 4. Materials and Methods

### 4.1. Cell Line Generation

RDEB keratinocytes and fibroblasts were isolated from a 3 mm skin biopsy of patients. HC-Kcs and fibroblasts were isolated from a healthy donor. Cells were E6/E7 immortalized and cultured under standardized conditions (37 °C and 5% CO_2_ in a humidified incubator) in CnT-Prime Epithelial Proliferation Medium (CELLnTEC, Bern, Switzerland) or in CnT Fibroblast Medium (CELLnTEC, Bern, Switzerland), respectively. 

### 4.2. ASO Synthesis

2′-MOE-modified oligonucleotides were obtained from Microsynth (Balgach, Switzerland). The sequences of all ASOs are shown in Supplementary Tables S1 and S2.

### 4.3. Constructs

Full-length *COL7A1*^c.425A>G^ was cloned by the Golden Gate cloning technique using NEBuilder^®^ Hifi DNA Assembly Cloning. The whole 9.2 kB cDNA was split into two fragments (F1: 4461 bp; F2: 4440 bp) and cloned into a pMXs-IRES-Blasticidin Retroviral vector (Cell Biolabs, San Diego, CA, USA) (F3: 5636 bp). Primers were designed using the NEBridge ^®^ Golden Gate Assembly Tool. Amplification of the fragments was performed using Phusion^TM^ High-Fidelity DNA Polymerase (ThermoFisher Scientific, Waltham, MA, USA) for F1 at an annealing temperature of 63.4 °C (forward primer: 5′-cctcgagggccggcgcgccgcATGACGCTGCGGCTTCTG-3′; reverse primer: 5′-cctttggggccAATAGCTCCAGGAGGTCCC-3′) and for F2 at an annealing temperature of 66.2 °C (forward primer: 5′-ctggagctattGGCCCCAAAGGTGACCGG-3′; reverse primer: 5′-gaggggcggaatttacgtagcTCAGTCCTGGGCAGTACCTG-3′). The vector was linearized with NotI restriction enzyme (New England Biolabs, Ipswich, MA, USA) and cloned according to the NEBuilder^®^ Hifi DNA Assembly Cloning (New England Biolabs, Ipswich, MA, USA) instructions.

### 4.4. Transfection

Transfection of RDEB keratinocytes with ASOs was performed using INTERFERin^®^ transfection reagent (Polyplus #101000028, Illkirch, France) according to the manufacturer’s protocol.

### 4.5. RNA Isolation

The RNA of ASO-transfected keratinocytes was isolated 48 h after treatment using an innuPREP RNA Mini Kit (Analytik Jena, Jena, Germany) and then transcribed into cDNA via the LunaScript RT SuperMix Kit (New England Biolabs, Ipswich, MA, USA) according to the manufacturer’s protocol. 

### 4.6. RT-PCR

RT-PCR for the detection of all alternative splice variants (ASV1–ASV4) was performed using GoTaq^®^ Polymerase (Promega, Madison, WI, USA). For the detection of the splice variants, the following specific primer set was used: exon 1/exon 2 junction forward primer (5′-CAGCACAGGGAGAGAGTGACCTGC-3′) and exon 4/exon 5 junction reverse primer (5′-GGGTCAGCATTCTTGATCCCCACAGC-3′). PCR products were analyzed on 3% agarose gel and confirmed by Sanger sequencing.

### 4.7. sqRT-PCR

sqRT-PCR was performed using the Luna^®^ Universal qPCR Master Mix (New England Biolabs, Ipswich, MA, USA) on a CFX96 Touch Real-Time PCR Detection System (BioRad, California, USA). As a reference gene, C7 exon 2 was used (forward primer: 5′-ATGACGCTGCGGCTTCTGG-3′; reverse primer: 5′-TTCGAGAAAGCTGCGGACCTCGCGGAAA-3′). To detect correctly spliced COL7A1, the forward primer was designed for C7 exon 1/2 (5′-CAGGGAGAGAGTGACCT-3′) and the reverse primer was mutation-specific for COL7A1 exon 3 (5′-GATCAGGATGCAGACCC-3′). PCR products were analyzed on 3% agarose gel and confirmed by Sanger sequencing.

### 4.8. Immunofluorescence Staining of C7 in Keratinocytes and Fibroblasts

A total of 72 h after transfection with ASOs, cells were fixed with 4% paraformaldehyde (PFA), blocked with blocking buffer (Roche Diagnostics GmbH, Mannheim, Germany) and then stained with a polyclonal anti-collagen type VII rabbit antibody or human-specific anti-collagen type VII antibody specific for the NC1 and NC2 domains [58], AlexaFluor^TM^ 488 (Thermo Fisher Scientific, Waltham, MA, USA), and DAPI (VWR, Vienna, Austria). Analysis was performed using an Axio Observer Z1 confocal laser scanning microscope attached to an LSM700 (Carl Zeiss, Berlin, Germany).

### 4.9. Densitometric Analysis of Splice Products via Fiji (ImageJ)

A grayscale image of the respective gel was opened in ImageJ (Fiji version number: ImageJ 2.1.0; Java 1.8.0_172) [58]. The first lane was highlighted with the rectangular selection tool and defined as “First Lane” (Strg + 1), while all subsequent lanes were defined as “Next Lane” (Strg + 2). After highlighting all lanes required for analysis, “Analyze->Gel->Plot Lanes” was used in order to obtain a profile for each lane. Afterwards, peaks were closed off with the straight line tool to remove the background, followed by measuring the peaks via the “Wand” tool. The values created by ImageJ were then incorporated into GraphPad Prim version 9.0.0 for Windows (GraphPad Software, San Diego, CA, USA) for further analysis.

### 4.10. Protein Isolation and Western Blot Analysis

For protein isolation, cells were lysed with a radioimmunoprecipitation assay (RIPA) buffer (Santa Cruz Biotechnology, Heidelberg, Germany) 72 h after transfection. After lysis, cells were centrifuged, and the clarified supernatant was used for analysis. Western blot analysis was performed as previously described [5]. Type VII collagen was detected using a polyclonal anti-collagen type VII rabbit antibody or human-specific anti-collagen type VII antibody specific for the NC1 and NC2 domains [59], and as a loading control, a β-tubulin-specific antibody (ab6064; Abcam, Cambridge, UK) was used. Goat anti-rabbit HRP-labelled antibody (Dako, Santa Clara, CA, USA) was used as a secondary antibody. Protein bands were visualized with the Immobilon Western Chemiluminescent HRP Substrate (Merck, Darmstadt, Germany) using the ChemiDoc XRS Imager (BioRad, Hercules, CA, USA). Native C7 Western blots were performed as previously described [60].

### 4.11. Generation of DDC642 Liposomes

DDC642 liposomes were generated according to the protocol of Desmet et al. [24]. Briefly, DOTAP (Sigma-Aldrich, St. Louis, MO, USA), DOPE (Sigma-Aldrich, St. Louis, MO, USA), and cholesterol (Sigma-Aldrich, St. Louis, MO, USA) were dissolved in chloroform (Sigma-Aldrich, St. Louis, MO, USA) and mixed in a ratio of 6 to 4.2 to 1.8. Chloroform was evaporated at 37 °C for approx. 4 h, resuspended with 1 mL 30% EtOH-HEPES, and incubated at room temperature in the dark for one day. The mixture was repeatedly extruded through a 100 nm polycarbonate membrane using a LiposoFast Factory (Sigma-Aldrich, St. Louis, MO, USA) and then stored at 4 °C.

### 4.12. Generation and Treatment of Skin Equivalents

A human fibrin scaffold was used for the generation of skin equivalents. A total of 5 × 10^4^ fibroblasts were immersed in a fibrinogen scaffold consisting of DMEM with 20% fetal cow serum (FCS); fibrinogen (F4883; Sigma-Aldrich, St. Louis, MO, USA) in 0.9% NaCl (final concentration = 25 mg/mL); thrombin (T8885; Sigma-Aldrich, St. Louis, MO, USA) dissolved in 25 mM CaCl_2_; and aprotinin (A6279; Sigma-Aldrich, St. Louis, MO, USA). The scaffold was directly prepared in Falcon^®^ permeable support inserts with a 0.4 µm transparent PET membrane (Corning, New York, NY, USA) and placed in BioCoat™ deep-well plates (6-well; Corning, New York, NY, USA) for 1 h at 37 °C and 5% CO_2_. A total of 5 × 10^5^ keratinocytes per well were seeded on top of the matrix and grown to confluence in CnT-Prime Epithelial Proliferation Medium (CELLnTEC, Bern, Switzerland). Wounding was performed by inserting a silicone cross on top of the keratinocyte monolayer. To allow stratification, skin equivalents were then raised to the air–liquid interface and cultured for 21 days. Before treatment, the silicone cross was removed and SEs were treated with a DDC642 liposome/ASO complex. For transfection, 10 µg of the respective ASO was complexed in a ratio of 1:16 with DDC642 liposomes (40 µg). After 10 min of incubation at room temperature, the complex was topically applied to the surface three times over the course of one week. Skin equivalents were harvested approximately 72 to 96 h after the last treatment, embedded in Tissue-Tek O.C.T. (Sakura Finetek USA, Inc., Torrance, CA, USA), and then processed for further analysis.

### 4.13. Immunofluorescence Staining of Skin Equivalents

Cryosections were fixed with acetone/methanol (1:1). Type VII collagen was detected with a human-specific anti-C7 antibody specific for the NC1 domain [59] and diluted 1:2000 in blocking buffer reagent (Roche Diagnostics GmbH, Mannheim, Germany). As a secondary antibody, Alexa Fluor^TM^ 488 goat anti-rabbit IgG (H + L) (ThermoFisher Scientific, Waltham, MA, USA) was used and diluted 1:400 in PBS together with 4′,6-Diamidin-2-phenylindol (DAPI) in a 1:2000 dilution (ThermoFisher Scientific, Waltham, MA, USA). Type XVII collagen detection was carried out using a rabbit polyclonal C-terminal antibody (184996) (Abcam, Cambridge, UK) diluted 1:500 in 1× blocking reagent from Roche diagnostics (Roche Diagnostics GmbH, Mannheim, Germany) diluted in PBS for 2 h at room temperature. After two washing steps with PBS, staining was performed with Alexa Fluor^TM^ 488 goat anti-rabbit immunoglobulin G (IgG; H + L) (1:400 in PBS) (ThermoFisher Scientific, Waltham, MA, USA) and 4′,6-diamidin-2-phenylindol (DAPI) (1:5000 in PBS) (ThermoFisher Scientific, Waltham, MA, USA) for 1 h at room temperature. As a secondary antibody, Alexa Fluor^TM^ 488 goat anti-rabbit IgG (H + L) (ThermoFisher Scientific) was used and diluted 1:400 in PBS together with 4′,6-Diamidin-2-phenylindol (DAPI) in a 1:2000 dilution (ThermoFisher Scientific). Cytokeratin 14 detection was carried out using a monoclonal mouse anti Cytokeratin 14 antibody (RCK107) (ab9220 Abcam, Cambridge, UK) diluted 1:200 in 1× blocking reagent from Roche diagnostics (Roche Diagnostics GmbH, Mannheim, Germany) diluted in PBS for 2 h at room temperature. As a secondary antibody, Alexa Fluor^TM^ 594 goat anti-mouse IgG (H + L) (Thermo Fisher Scientific, Waltham, MA, USA) was used and diluted 1:400 in PBS together with 4′,6-Diamidin-2-phenylindol (DAPI) in a 1:2000 dilution (ThermoFisher Scientific, Waltham, MA, USA).

Finally, cryosections were washed with PBS, H_2_O, and 100% ethanol, covered with a DAKO fluorescent mounting medium (Agilent, Santa Clara, CA, USA), and analyzed using an Axio Observer Z1 confocal laser scanning microscope attached to an LSM700 (Carl Zeiss, Berlin, Germany).

### 4.14. Immunohistochemistry Staining of Skin Equivalents

Cryosections were also used for hematoxylin and eosin (H&E) staining. After washing the slides with H_2_O (30 dips), they were stained with Mayer’s hemalum solution (Merck, Kenilworth, NJ, USA) for 6 min. After a second washing step with 30 dips in H_2_O, slides were dipped 10 times in 0.3% HCl/EtOH solution and 30 times in H_2_O and then incubated for 2 min in 0.5% Eosin G solution (Merck, Kenilworth, NJ, USA). Slides were then washed with 30 dips in H_2_O, followed by 30 dips in isopropanol and 30 dips in HistoChoice^®^ Clearing Agent (Merck, Kenilworth, NJ, USA). Finally, cryosections were covered with ROTI^®^Histokitt (CarlRoth, Karlsruhe, Germany) and stored at 4 °C. Samples were analyzed using an Axio Observer Z1 confocal laser scanning microscope attached to an LSM700 (Carl Zeiss, Berlin, Germany) and a VS120 automated slide scanner (Olympus, EVIDENT Europe GmbH, Hamburg, Germany). 

### 4.15. ddPCR

ddPCR reactions were prepared according to the manufacturer’s instructions, using the 2X ddPCR super-mix without dUTP (BioRad, Hercules, CA, USA). Primers and probes were designed according to the ddPCR mutation detection assay from BioRad, with a primer-to-probe ratio of 3.6 (the primer concentration in the final reaction was 900 nM and the probe concentration was 250 nM). As a reference gene, C7 exon 2 was used (forward primer: 5′-TGACATTGTGTTCTTACTGG-3′; reverse primer: 5′-CTCGCGGAAATTGCTG-3′) with an HEX-labeled probe (5′-TGGCTCCTCATCCATTGG-3′). For amplification of C7 ASV2, the following primers were used: forward primer in exon 3: 5′-CCAGCTGGCCCGA-3′; reverse primer in exon 4: 5′-GACTTCCCGTCTGTGAT-3′ together with a mutation-specific FAM-labeled probe (5′-TGTCCCCAGGGTCTG-3′). After generation of droplets in the BioRad QX200 generator, a 2-step cycling protocol was used for 10 min at 95 °C, followed by 40 cycles of 30 s at 94 °C and 1 min at 56 °C with a ramp of 1.5 °C/s and a final denaturation at 98 °C for 10 min. Subsequently, ddPCR reactions were analyzed in a BioRad QX200 droplet reader and data analysis was performed with BioRad’s QX Manager Software 2.0.

### 4.16. TECAN Spark^®^ Measurement

To verify successful nuclear localization of ASOs, treated cells were analyzed with the TECAN Spark^®^ (Tecan Group Ltd., Männedorf, Switzerland) Cyto plate reader’s imaging module (10× maginification) to detect fluorescence signal from both Cy5-labeled ASOs and Hoechst33342 (NucBlue™ Live ReadyProbes™ Reagent, ThermoFisher Scientific, Waltham, MA, USA)-stained nuclei. 

### 4.17. Statistics

Unpaired Student’s t-tests as well as a one-way ANOVA (with the appropriate multiple comparisons test) were performed using GraphPad Prism (GraphPad Prism version 9.0.0 for Windows, GraphPad Software, San Diego, CA, USA). *p*-values (significances): >0.05 (not significant), ≤0.05 (*), ≤0.01 (**), ≤0.001 (***), ≤0.0001 (****).

## Figures and Tables

**Table 1:** RDEB keratinocyte and fibroblast patient cell line mutations.

Cell Line	Mutation	Exon	Cell Type
RDEB1	c.425A > G/c.6841G > T	exon 3, exon 63	keratinocytes
RDEB2	c.425A > G/c.520G > A	exon 3, exon 4	keratinocytes
RDEB3	c.425A > G/c.520G > A	exon 3, exon 4	keratinocytes
RDEB4	c.425A > G/c.4027C > T	exon 3, exon 34	keratinocytes
RDEB5	c.425A > G/c.425A > G	exon 3, exon 3	keratinocytes
RDEB5	c.425A > G/c.425A > G	exon 3, exon 3	fibroblasts
RDEB6	c.425A > G/c.682 + 5G > A	exon 3, intron 5	keratinocytes
RDEB7	c.425A > G/c.425A > G	exon 3, exon 3	keratinocytes
RDEB8	c.425A > G/c.425A > G	exon 3, exon 3	keratinocytes

## Data Availability

All data maintained in the course of this study are available within the manuscript and within its supplementary materials.

## Supplementary Materials

The following supporting information can be downloaded at: https://www.mdpi.com/article/10.3390/ijms25020761/s1.
