# Supplementary material for: Splicing Modulation via Antisense Oligonucleotides in Recessive Dystrophic Epidermolysis Bullosa
Source: Int J Mol Sci. 2024 Jan 7;25(2):761. doi: 10.3390/ijms25020761 (PMC10815346; doi:10.3390/ijms25020761)
Supplement: Supplementary file 1 [file ijms-25-00761-s001.zip › ijms-2777521-supplementary.pdf]

International Journal of
Molecular Sciences

MDPI

Supplementary Figures:

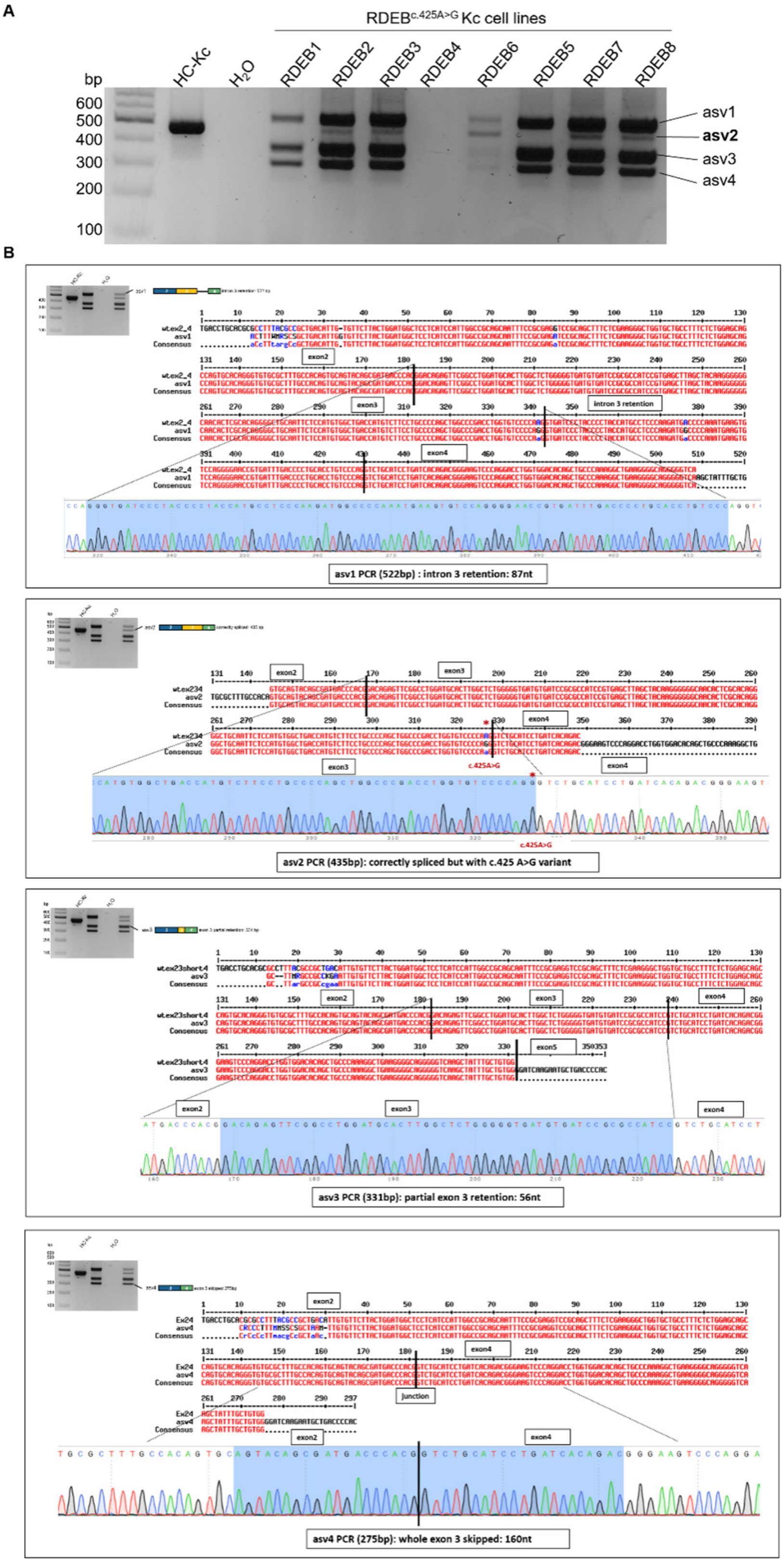

Supplementary Figure 1: (A) Residual levels of correctly-spliced *COL7A1* (asv2) were detected in 6 out of 8 primary keratinocytes from RDEB patients carrying the homozygous c.425A>G (p.K142R) variant on genomic level. **(B)** Sequence analysis of all detected splice variants.

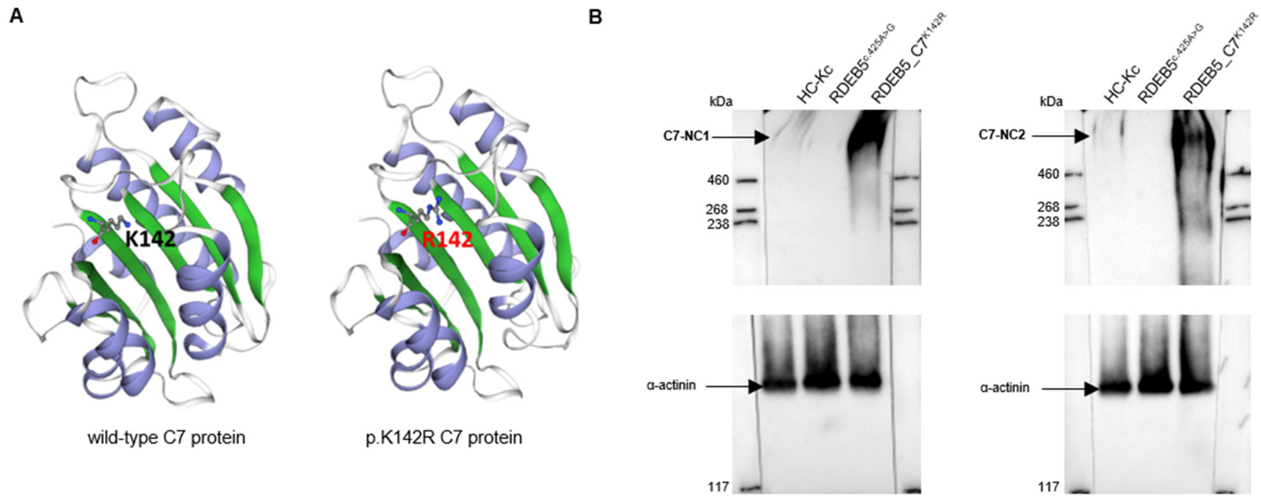

Supplementary Figure 2: (A) Prediction of protein folding of wildtype C7 protein (left) and C7 protein carrying a single conservative amino acid exchange (p.K142R). Modeling was done on SWISS-MODEL Interactive Workspace (expasy.org) using the von Willebrand Factor 1A domain (D38-V211) of C7. Ball-and-stick representation of lysine (K) and arginine (R) at position 142 is shown. **(B)** C7 trimer detection in RDEB5_C7$^{p.K142R}$ Kc using native Western Blot conditions. Cell lysates were separated on 7 % Tris-Acetate gels. α-Actinin was used as loading control.

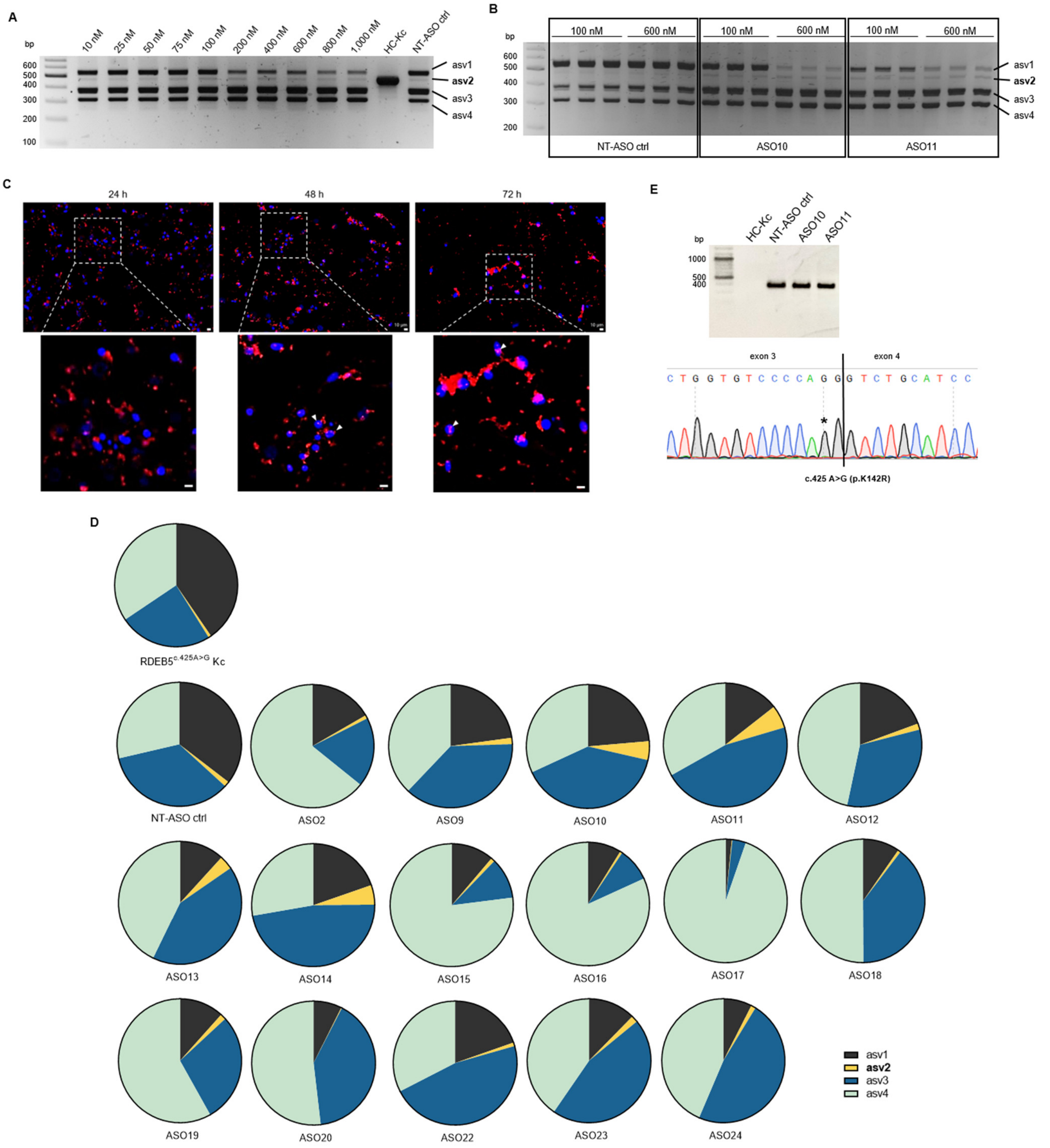

A
bp
10 nM
25 nM
50 nM
75 nM
100 nM
200 nM
400 nM
600 nM
800 nM
1,000 nM
HC-Kc
NT-ASO ctrl
600
500
400
300
200
100
asv1
asv2
asv3
asv4
B
100 nM
600 nM
NT-ASO ctrl
ASO10
ASO11
C
24 h
48 h
72 h
E
HC-Kc
NT-ASO ctrl
ASO10
ASO11
1000
exon 3
exon 4
C T G G T G T C C C C A G G G T C T G C A T C C
c.425 A>G (p.K142R)
D
RDEB5c.425A>G Kc
NT-ASO ctrl
ASO2
ASO9
ASO10
ASO11
ASO12
ASO13
ASO14
ASO15
ASO16
ASO17
ASO18
ASO19
ASO20
ASO22
ASO23
ASO24

Supplementary Figure 3: (A) Initial concentration kinetics analysis to determine the concentration of ASO necessary to obtain the highest amount of asv2. **(B)** Second concentration kinetics analysis of the NT-ASO control, ASO10 and ASO11 to highlight the increased amount of asv2 when treated with 600 nM. **(C)** After transfection of RDEB5^{K142R} cells with ASO11-Cy5, accumulation of ASOs was detectable in nuclei already 24 h post transfection, with a peak in signal intensity at 48 h. After 72h increased cell death was detectable. Cell nuclei were counterstained with Hoechst 33342 Solution, 20 mM (Thermo Fisher). Scale bar = 10 µm. **(D)** Depicturing of the amount of asv1, asv2, asv3 and asv4 in RDEB5^{K142R} Kc after ASO transfection. **(E)** Representative analysis of the specificity of the sqPCR assay to specifically detect the asv2 transcript out of all *COL7A1*-transcripts present. Sanger sequencing confirmed the clean amplification of asv2. Densitometric analysis n = 3; sqPCR analysis n = 5.

Supplementary Tables:

Supplementary Table 1: Sequences of all ASOs tested in the macro-walk.

Macro-walk		
**ASO**	**Sequence**	**% G~C content**
neg ASO	T*G*T*G*G*C*G*A*G*T*A*G*A*C*T*C*G*A*A*G	55%
ASO 2	G*C*C*G*A*A*C*T*C*T*G*T*C*C*T*G*T*T*G*G	60%
ASO 9	G*G*G*T*C*A*T*C*T*T*G*G*G*A*G*G*C*A*T*G	60%
ASO 10	A*C*G*G*T*T*C*C*C*C*T*G*G*A*C*A*C*T*T*C	60%
ASO 11	C*T*G*G*G*A*C*A*G*G*T*G*C*A*G*G*G*G*T*C	70%
ASO 12	A*C*A*C*T*T*C*A*T*T*T*G*G*G*G*T*C*A*T*C	45%
ASO 13	C*A*G*G*G*G*T*C*A*A*A*T*C*A*C*G*G*T*T*C	55%
ASO 14	T*C*A*G*G*A*T*G*C*A*G*A*C*C*T*G*G*G*A*C	60%
ASO 15	C*A*T*C*C*A*G*G*C*C*G*A*A*C*T*C*T*G*T*C	60%
ASO 16	C*C*C*C*A*G*A*G*C*C*A*A*G*T*G*C*A*T*C*C	65%
ASO 17	C*G*C*G*G*A*T*C*A*C*A*T*C*A*C*C*C*C*C*A	65%
ASO 18	T*A*A*G*C*T*C*A*C*G*G*A*T*G*G*C*G*C*G*G	65%
ASO 19	T*G*C*C*C*C*C*C*T*T*G*T*A*G*C*T*A*A*G*C	60%
ASO 20	C*C*C*C*T*G*T*G*C*G*A*G*T*G*T*T*G*C*C*C	70%
ASO 21	C*A*T*G*G*A*G*A*A*T*T*G*C*A*G*C*C*C*C*T	55%
ASO 22	A*G*A*C*A*T*G*G*T*C*A*G*C*C*A*C*A*T*G*G	55%
ASO 23	C*C*A*G*C*T*G*G*G*G*C*A*G*G*A*A*G*A*C*A	65%
ASO 24	G*G*A*C*A*C*C*A*G*G*T*C*G*G*G*C*C*A*G*C	75%

* phosphorothioate bonds

Supplementary Table 2: Sequences of all ASOs tested in the micro-walk.

Micro-walk		
**ASO**	**Sequence**	**% G~C content**
ASO 10.1	G*G*T*T*C*C*C*C*T*G*G*A*C*A*C*T*T*C*A*T	55%
ASO 10.2	C*G*G*T*T*C*C*C*C*T*G*G*A*C*A*C*T*T*C*A	60%
ASO 10.3	A*C*G*G*T*T*C*C*C*C*T*G*G*A*C*A*C*T*T*C	60%
ASO 10.4	C*A*C*G*G*T*T*C*C*C*C*T*G*G*A*C*A*C*T*T	60%
ASO 10.5	T*C*A*C*G*G*T*T*C*C*C*C*T*G*G*A*C*A*C*T	60%
ASO 10.6	A*T*C*A*C*G*G*T*T*C*C*C*C*T*G*G*A*C*A*C	60%
ASO 10.7	A*A*T*C*A*C*G*G*T*T*C*C*C*C*T*G*G*A*C*A	55%
ASO 10.8	A*A*A*T*C*A*C*G*G*T*T*C*C*C*C*T*G*G*A*C	55%

ASO 11.1	C*A*A*A*T*C*A*C*G*G*T*T*C*C*C*C*T*G*G*A	55%
ASO 11.2	T*C*A*A*A*T*C*A*C*G*G*T*T*C*C*C*C*T*G*G	55%
ASO 11.3	G*T*C*A*A*A*T*C*A*C*G*G*T*T*C*C*C*C*T*G	55%
ASO 11.4	G*G*T*C*A*A*A*T*C*A*C*G*G*T*T*C*C*C*C*T	55%
ASO 11.5	G*G*G*T*C*A*A*A*T*C*A*C*G*G*T*T*C*C*C*C	60%
ASO 11.6	G*G*G*G*T*C*A*A*A*T*C*A*C*G*G*T*T*C*C*C	60%
ASO 11.7	A*G*G*G*G*T*C*A*A*A*T*C*A*C*G*G*T*T*C*C	55%
ASO 11.8	C*A*G*G*G*G*T*C*A*A*A*T*C*A*C*G*G*T*T*C	55%
ASO 11.9	G*C*A*G*G*G*G*T*C*A*A*A*T*C*A*C*G*G*T*T	55%
ASO 1.10	T*G*C*A*G*G*G*G*T*C*A*A*A*T*C*A*C*G*G*T	55%
ASO 1.11	G*T*G*C*A*G*G*G*G*T*C*A*A*A*T*C*A*C*G*G	60%
ASO 1.12	G*G*T*G*C*A*G*G*G*G*T*C*A*A*A*T*C*A*C*G	60%
ASO 11.13	A*G*G*T*G*C*A*G*G*G*G*T*C*A*A*A*T*C*A*C	55%
ASO 11.14	C*A*G*G*T*G*C*A*G*G*G*G*T*C*A*A*A*T*C*A	55%
ASO 11.15	A*C*A*G*G*T*G*C*A*G*G*G*G*T*C*A*A*A*T*C	55%
ASO 11.16	G*A*C*A*G*G*T*G*C*A*G*G*G*G*T*C*A*A*A*T	55%
ASO 11.17	G*G*A*C*A*G*G*T*G*C*A*G*G*G*G*T*C*A*A*A	60%
ASO 11.18	G*G*G*A*C*A*G*G*T*G*C*A*G*G*G*G*T*C*A*A	65%
ASO 11.19	T*G*G*G*A*C*A*G*G*T*G*C*A*G*G*G*G*T*C*A	65%
ASO 11.20	C*C*T*G*G*G*A*C*A*G*G*T*G*C*A*G*G*G*G*T	70%
ASO 11.21	A*C*C*T*G*G*G*A*C*A*G*G*T*G*C*A*G*G*G*G	70%
ASO 11.22	G*A*C*C*T*G*G*G*A*C*A*G*G*T*G*C*A*G*G*G	70%
ASO 11.23	A*G*A*C*C*T*G*G*G*A*C*A*G*G*T*G*C*A*G*G	65%

* phosphorothioate bonds
